# Mediterranean Diet a Potential Strategy against SARS-CoV-2 Infection: A Narrative Review

**DOI:** 10.3390/medicina57121389

**Published:** 2021-12-20

**Authors:** Yvelise Ferro, Roberta Pujia, Samantha Maurotti, Giada Boragina, Angela Mirarchi, Patrizia Gnagnarella, Elisa Mazza

**Affiliations:** 1 Department of Medical and Surgical Science, University Magna Græcia, 88100 Catanzaro, Italy; roberta.puj@gmail.com (R.P.); smaurotti@unicz.it (S.M.); angy551@hotmail.it (A.M.); elisamazza@unicz.it (E.M.); 2 Department of Clinical and Experimental Medicine, University Magna Græcia, 88100 Catanzaro, Italy; giadyboragina@gmail.com; 3 Division of Epidemiology and Biostatistics, IEO European Institute of Oncology IRCCS, 20141 Milan, Italy; patrizia.gnagnarella@ieo.it

**Keywords:** Mediterranean diet, COVID-19, inflammation, nutrition, SARS-CoV-2

## Abstract

Mediterranean Diet represents the traditional eating habits of populations living around the Mediterranean Sea, and it is associated with a lower risk of overall mortality and cancer incidence and cardiovascular diseases. Severe acute respiratory syndrome coronavirus 2 is a new pandemic, and represents a significant and critical threat to global human health. In this study, we aimed to review the possible effects of Mediterranean Diet against the risk of the coronavirus disease 2019. Several vitamins, minerals, fatty acids, and phytochemicals with their potential anti-COVID-19 activity are presented. Different risk factors may increase or reduce the probability of contracting the disease. Mediterranean Diet has also a positive action on inflammation and immune system and could have a protective effect against severe acute respiratory syndrome coronavirus 2. Further studies are needed to corroborate the benefits of the Mediterranean Diet protective role on infection with SARS-CoV-2.

## 1. Introduction

The first part of 2020 was characterized by the pandemic spread of a novel coronavirus: severe acute respiratory syndrome coronavirus-2 (SARS-CoV-2) [1]. To date, millions of cases of coronavirus disease 19 (COVID-19) have been reported, and it has caused more than 3.9 million deaths in the world [2]. The population generally is not provided with immunity to SARS-CoV-2 and is susceptible to the new virus disease [1,3,4,5]. Previous epidemiological and clinical studies featuring COVID-19 have shown that SARS-CoV-2 infection usually results in mild disease, although several patients occasionally develop severe or critical illness [1,3,4,5,6,7,8]. In particular, asymptomatic individuals are estimated to range from 17.9% to 78% [3], approximately 15% of infected individuals will develop severe illness, and about 5% will eventually develop severe pneumonia and acute respiratory distress syndrome (ARDS) [4]. Some COVID-19 patients will also develop systemic manifestations such as sepsis, cardiovascular complications, thromboembolism, and multi-organ failure [4,8].

Worsening clinical outcomes of COVID-19 have been associated with older age, male gender, and the presence of comorbidities such as hypertension, obesity, and type 2 diabetes mellitus [5,6,7].

A recent report shows data from the COVID-19 case fatality rate (CFR) in Italy, highlighting a linear relationship between the CFR and age [9]. In particular, CFRs are less than 0.4% in patients aged 40 or younger, 1% among those aged 50, 3.5% in those aged 60, 12.8% in those aged seventy, at 20.2% in those over eighty; the overall CFR is 7.2% [9]. Recent evidence suggests that the SARS-CoV-2 viral loads are similar in asymptomatic, mild symptomatic, and severe symptomatic patients [10,11], but many other factors influence the progression and severity of the disease.

Currently, no specific data have been reported regarding the immunological response to SARS-CoV-2, but new studies have shown that a cytokine storm overstimulates the body’s immune response to microorganisms as a consequence of increases in the levels of inflammatory factors [12]. Therefore, the inflammatory factors contribute to one of the most important mechanisms underlying disease progression and death. Then, the coexistence of both COVID-19 and chronic diseases should be considered alarming, because it represents the combination of more pandemics [13]. The interaction between nutrition, immune function, inflammation, and infection represents a key tool to reduce the risk of susceptibility and morbidity of viral infectious diseases [13,14,15]. Research has shown that greater adherence to the Mediterranean diet (MetDiet) is associated with a reduced risk of major chronic diseases, [16] due to its anti-inflammatory and immune-modulatory properties. Thus, we hypothesize that the MetDiet could play a potentially beneficial role in people with SARS-CoV-2 infection.

The findings presented in this paper should promote nutritional information on the positive effects of the Mediterranean Diet against the risk of COVID-19.

## 2. The Mediterranean Diet: A Healthy Dietary Pattern for People with SARS-CoV-2 Infection

The MetDiet is a model of eating based on the traditional foods and drinks of the countries surrounding the Mediterranean Sea. Over the last few decades, this nutritional model has been promoted worldwide as one of the healthiest dietary patterns and has been reported to be consistently beneficial with regard to longevity. The MetDiet is characterized by high consumption of unrefined cereals, fruit, vegetables, legumes, and olive oil, moderate consumption of dairy products and wine, and low meat consumption [16,17].

Among other benefits, adhering to the MetDiet has been linked to a lower risk of various chronic conditions [18,19,20,21,22], with lower risk of inflammation as well as increased immunity [23,24]. Its protective properties are thought to be a combination of the high intake of polyunsaturated fatty acids (PUFA) from fish [25], monounsaturated fatty acids (MUFA) and polyphenols from extra virgin olive oil (EVOO) [26], and antioxidants from fruit, vegetables, legumes, and wine [20,26,27]. Furthermore, the MetDiet is rich in phytochemicals with antioxidant action, minerals, and vitamins [23].

The first umbrella review meta-analysis of observational studies and randomized trials estimated the association between adherence to the MetDiet and 37 different health outcomes, including overall mortality, cardiovascular and cancer outcomes, neurodegenerative and metabolic disorders, as well as inflammatory markers. This meta-analysis showed that a greater adherence to the MetDiet reduced the risk of overall mortality and cancer incidence, cardiovascular and neurodegenerative diseases, and diabetes [16].

Each component of the MetDiet has its benefits, but it can be assumed that it is the combination of various nutrients that is the basis of the extraordinary health effects of MetDiet [16,17], especially on the immune system [28,29].

Recent research showed that one MetDiet-style meal reduced the expression of pro-inflammatory molecules [29], the overall systemic inflammatory status [30], and several diseases associated with chronic low-grade inflammation. In adult individuals, a MetDiet intervention led to lower glycoxidative impairment [31] and inflammatory response [32,33]. A meta-analysis including 2300 subjects reported a significant reduction in high-sensitivity C-reactive protein (hs-CRP) (−0.98 mg/L, *p* < 0.0001), intracellular adhesion molecule-1 (−23.73 ng/mL, *p* = 0.008), and IL-6 (−0.42 pg/mL, *p* = 0.008) in individuals assigned to MetDiet, compared with those following a control intervention protocol [34].

A potential protection against COVID-19 by a MetDiet was assessed longitudinally in a cohort of 5194 non-health professionals [35]. Participants with the highest adherence to MetDiet had a significantly lower odds of developing SARS-CoV-2 infection compared with those with lowest adherence (multivariable-adjusted OR = 0.36, 95% CI: 0.16–0.84; *p* for trend < 0.001) [35].

An ecological study, of only European countries, showed a significant negative association between MetDiet and COVID-19-related deaths (r^2^ = 0.771, *p* = 0.030) [36]. The authors observed that MetDiet adherence was negatively associated with COVID-19 cases as well as related deaths across 17 regions in Spain and that the relationship remained also after adjustment for factors of well-being [36]. The same authors also observed a negative association between Metdiet adherence and COVID-19-related deaths across 23 countries (OECD) after adjustment for physical inactivity and some confounding factors [36].

An observational case control study explored the possible associations among different dietary patterns and COVID-19 events and outcomes. The results showed that the cases had a lower mean of the MedDiet score (*p* = 0.009) than controls did, demonstrating an inverse association between the MetDiet and COVID-19 risk [37].

The preliminary results of an experimental study aimed to detect the beneficial effects of MetDiet before and after the period of COVID-19 Lockdown in Mediterranean area (Spain) old individuals showed that patients who initiated the MetDiet intervention program before Lockdown increased their level of adherence to the MetDiet by 3.5% and maintained an adequate nutritional status after the Lockdown. In the BMI, there no were statistically significant differences between experimental and control groups before and after Lockdown. These results suggest that adherence to the MetDiet may play an important role in the maintenance of an adequate nutritional status in the confinement situations such as the COVID-19 Lockdown [38].

**Figure 1:**
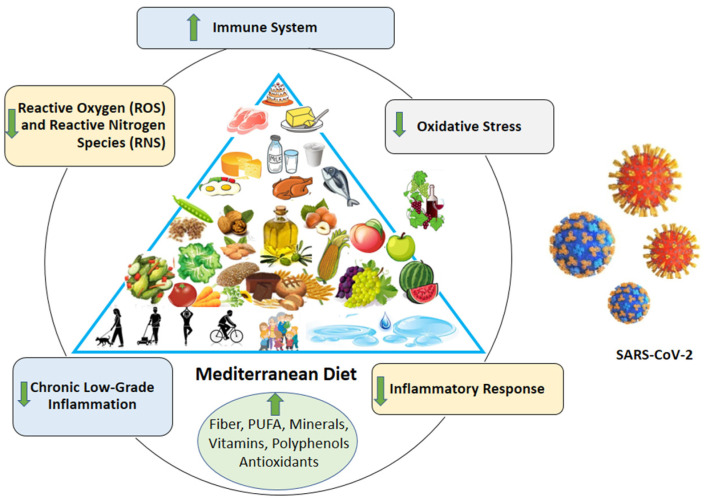
Mediterranean diet: potential strategy against coronavirus infection.

All these results suggest the important role that nutrition, and, in particular, the MetDiet, could play in the prevention and management of COVID-19 infection (Figure 1).

## 3. Mediterranean Diet and COVID-19: Plausible Mechanisms of Potential Benefits

COVID-19 is characterized by increased levels of numerous cytokines, mainly of proinflammatory character, including tumor necrosis factor-alpha (TNF-alpha), interleukin-6 (IL-6), and CRP [39]. Therefore, effective treatment strategies pursued could include reducing inflammation in order to prevent the risk of infection or blunt the severity of the COVID-19 disease [12]. In this regard, several studies suggest that MetDiet induces positive effects on both inflammation and oxidative stress. The stimulating effect induced at the level of the immune system is pointed out by the positive results induced by MetDiet on people with inflammatory phenomena impacting the respiratory system [40]. Several micronutrients have been suggested to act as immunomodulatory agents against COVID-19, and they are briefly summarized in Table 1.

Fruits, whole grains, vegetables, fish, PUFA, and MUFA have been found to cause less inflammation in the body [41], while foods with high saturated fat content such as processed red meat, cheese, and dairy may induce inflammation [40]. It may be the abundance of beneficial foods (rich in fiber, PUFA, minerals, vitamins, polyphenols, and antioxidants) and lack of fatty foods (rich in starch, refined sugar and trans fatty acids) in the MetDiet that produce its favorable effects [42].

PUFAs include long-chain omega-3 PUFAs, EPA (20:5n–3), and DHA (22:6n–3), derived mainly from fish and seafood [41], as well as α-linolenic acid, derived from various plant sources [43]. Among PUFAs, the omega-3 free fatty acids exert anti-inflammatory effects via specialized pro-resolving mediators, which are the oxylipins, of oxygenated metabolites [25,44].

Dietary fibers are an important factor regarding the influence of complex carbohydrates on inflammation [45,46]. It was demonstrated that an increase in fiber consumption (about 30 g/d) was associated with a significant reduction in hs-CRP concentrations [47]. Another advantage of dietary fiber intake is a more favorable gut microbiome composition, which lowers both gut and systemic inflammation, and even small increases of fiber (5 g/d) can be beneficial [89,90]. Watanabe et al. hypothesized that a rice-eating habit seems to be a factor that explains the reason for low COVID-19 incidence and mortality in rice-eating countries. The authors make a hypothesis that populations who consume rice have a special profile of microbiota that produce butyrate, which stimulates the proliferation of regulatory T cells, prevents a cytokine storm (induced by the infection), and reduces the levels of IL-6 and CRP [91].

Although it is the most consumed food in Asia, rice plays a key role also in the diet of many countries, including those of the Mediterranean area [92].

Modifications in the intestinal barrier contribute to the pathogenesis of many illnesses; viruses may also contribute in disrupting the intestinal epithelium [93]. Sharma clarified that the gastrointestinal structure and the gut barrier may be affected by SARS- CoV-2 virus, and disorder of barrier functions or intestinal microbial dysbiosis may influence the progression and severity of COVID-19 disease [93]. It has been shown that the SARS-CoV-2 virus can impact PALS1, a tight junction-associated protein, present in the intestinal and lung epithelium [71]. For this, it has been proposed that SARS-CoV-2 may increase intestinal permeability, causing damage to enterocytes and the epithelial layer [72].

MetDiet is also very rich in prebiotic substances, such as galactans, fructans, fibers, and inulins. Numerous reports indicate that these compounds are used by host microorganisms, supporting the growth of favorable bacteria and by promoting the production of beneficial metabolites [48,49,93].

There is also evidence supporting the protective role of vitamins against viral infections through multiple mechanisms [44]. EVOO is one of the staple foods of the MetDiet, and is the main dietary source of vitamin E. This vitamin is one of the most effective nutrients enhancing immune function and inflammation [44,50]. Several studies have indicated that vitamin E deficiency impairs both humoral and cell-mediated immune functions [51,52]. Vitamin E and vitamin C are well-known antioxidant compounds, able to reduce the production of reactive oxygen species (ROS) and reactive nitrogen species (RNS) [44,73]. Moreover, Vitamin A is involved in the production of mucin secretion and enhancing antigen nonspecific immunity functions (healthy mucus stratum), such as those of the bowel and the respiratory tract [51,52].

Many studies have highlighted the ability of vitamin D to reduce infections and to modulate innate and adaptive cellular immunity, and have shown an inverse association between the incidence of airway infections and its serum levels [74].

Furthermore, vitamin D administration has also been reported to provide protective effects regarding the incidence and severity of influenza [75]. The use of vitamin D to reduce the severity of SARS-CoV-2 complications is receiving remarkable attention. It was show that vitamin D facilitates the binding of the SARS-CoV-2 cell entry receptor angiotensin-converting enzyme 2 (ACE2) to angiotensin II receptor type 1 (AGTR1), decreasing the number of viral particles that could attach to ACE2 and enter the cell [12,44]. However, many aspects related to vitamin D are still to be clarified.

Among the specific minerals of the MetDiet, zinc is an essential trace element, and its impact on immune system has been a topic of intensive study [76]. An inadequate zinc intake has been reported to be associated with increased probability of viral infections [77]. In particular, zinc, in its free form (unchelated), has been associated with an immediate antiviral effect [78]. A significant percentage of the elderly have low serum zinc levels due to inadequate intake, infection, inflammation, etc. [53]. Shellfish, beef, nuts, and legumes are good sources of zinc [53].

A recent review emphasized the association between low zinc condition and pneumonia in the elderly. Specifically, death due to pneumonia has been reported to be twice as high in elderly subjects with low zinc levels compared to those with normal zinc levels. Inadequate stores of zinc might, therefore, be a risk factor for pneumonia in the elderly [53]. For this reason, it has been recommended that zinc may lessen common cold symptoms.

The MetDiet is also a source of large amounts of selenium [79]. The content of selenium in foods is characterized by a great variability depending on different factors (climatic conditions, concentration in the soil, cultivation and breeding methods, and methods of preparing food products), and fish, meat, offal, dairy, and cereals are good sources of selenium [53]. The impact of selenium on immune functions and underlying molecular mechanisms was discussed recently [80]. Furthermore, the relationship between selenium and influenza virus has been demonstrated [81], as well as its role as an adjuvant therapy in viral infections [80]. Selenium deficiencies have been associated with influenza infections, determining adaptive and innate immunity responses and leading to a high level of virus-related pathogenicity. Selenium’s primary role is its ability as an antioxidant to quench ROS [82]. It has also been reported that selenium is protective against effects of the cytomegalovirus [83], and is involved in immunoglobulin production and in T-lymphocyte proliferation [83,84]. Recently, the combined deficit of zinc and selenium was found in patients with COVID-19 at admittance to hospital [84]. The important deficits observed for both minerals in samples from newly admitted patients with COVID-19 point to an interfering and robust disrupting action of the virus on basic metabolic routes for these two essential elements [54]. Therefore, following a diet rich in these micronutrients, such as the MetDiet, could improve the outcome of SARS-CoV-2 infection.

Platelet-activating factor (PAF) is an important molecule implicated in COVID-19 pathology, as a potent mediator of inflammation and thrombosis [55,85]. Several micronutrients of MetDiet, such as vitamins A, C, E, and D, selenium, zinc, phytochemicals, and omega-3 PUFAs, have potential antithrombotic and anti-PAF effects, and they could act as immunomodulatory agents against COVID-19 [56].

The MetDiet, with is high intake of vegetables and fruits, especially those rich in flavonoids, significantly reduced serum inflammatory markers (IL-6 and CRP) and adhesion factors [57,58]. A lot of flavonoids, including quercetin, have been studied in vitro, to examine their potential antiviral effects: replication and infectivity of viruses, such as parainfluenza virus type 3 (Pf-3) and respiratory syncytial virus (RSV) [59]. Likewise, quercetin prevented intracellular viral replication and decreased viral infectivity, depending on its concentration, when cell cultures were infected and afterwards cultured in quercetin-containing medium [59].

Polyphenols are among the most abundant secondary plant compounds or phytochemicals in the MetDiet [94] and likely exert numerous antioxidant and anti-inflammatory effects [86,95] through inhibition of NF-κB and AP-1 and activation of Nrf2 [87].

Polyphenols are said to possess prebiotic effects on the gut microbiota [60,88]. The role of polyphenols against influenza viruses has been reconsidered recently [61]. A strong anti-influenza virus activity, in cell and in mice models, was shown following the administration of an extract rich in polyphenols [62]. In various cell models, coumarin, a non-flavonoid polyphenol, was shown to have anti-influenza activity and positive effect against viral infections, such as of HIV, influenza, and coxsackievirus A16 [63]. Polyphenols, present in black tea, showed a strong inhibitory effect against the influenza virus in vitro [88], probably due to their downregulation effect of IL-6 expression.

Growing evidence from in vivo and in vitro experiments suggests that resveratrol, a polyphenolic compound contained in the MetDiet, may influence ACE2 expression [64], protecting against age-related vascular diseases and reducing cardiovascular risk in the elderly population. Marlies de Ligt investigated the effects of resveratrol supplementation for 1 month (150 mg/day) in males with obesity, otherwise healthy (not using medications and no family history of diabetes or any other disorder), in a randomized, placebo-controlled cross study, which showed that resveratrol reduced ACE2 (~40%) and leptin (~30%), which could reduce the spread of SARS-CoV-2 [65], rendering them less susceptible for SARS-CoV-2 via lower ACE2 receptor expression in adipose tissue.

Other polyphenol constituents seem to shown a similar antiviral effect [66]. Studies showed that phenolic compounds present in MetDiet are able to inhibit the SARS-CoV-2 virus, through a competitive linkage, hindering the access of the virus into cells [67]. Therefore, following the MetDiet style brings a myriad of polyphenols and antioxidants, with beneficial effects on the progression of SARS-CoV-2.

Recent scientific evidence showed that some antioxidant molecules, particularly tannins, may exert prebiotic-like effects. They are a heterogeneous group of polyphenolic compounds present in numerous foods (cereals, fruits, and legumes) and responsible for the astringent taste of many fruits and vegetables. It seems that they can promote the growth of Bifidobacteria and Lactobacilli [68,69], which play a key role in regulating immune responses [70]. Therefore, MetDiet could modulate the ecology of gut microbiota to enable a balanced immune response against SARS-CoV-2 [70].

No single food has the potential to prevent or treat coronavirus, but numerous foods and nutrients included in the Mediterranean diet pattern could positively influence the outcomes of SARS-CoV-2 infection. This topic is of growing interest to researchers, the general population, and the media. Certain nutrients, such as vitamin D, vitamin C, and selenium, have attracted attention, mainly due to a deficient status linked to the severity of SARS-CoV-2 infection and COVID-19 disease. However, the potential benefit of foods and nutrients supplementation as a protective measure against these conditions remains a controversial topic. Therefore, the diet adopted by the population plays a decisive role, in order to integrate all potentially beneficial nutrients [102]. To this end, following MetDiet could be a useful strategy to achieve these goals.

A comparative study among a country (Spain) associated with a MetDiet and other countries with less adherence to MetDiet showed that subjects with greater MetDiet adherence could be better protected from harm caused by SARS-CoV-2, especially in subjects more susceptible to severe symptoms of COVID-19, such as the obese population. [67,96,97]. MetDiet has been associated with beneficial effects on body weight, visceral fat, blood pressure, and blood lipids, conditions associated with the severity of COVID-19 disease [98].

In addition to its favorable impact on overall mortality, cardiovascular and cancer outcomes, and neurodegenerative and metabolic disorders, the anti-inflammatory effects of the MetDiet have been recently explored, due to the whole dietary pattern or to its main components. These anti-inflammatory effects are considered to provide health benefits for older people [99], and play a role in bone mineralization, which is particularly important due to the reduction of physical activity and mobility in lockdown situations.

Although amelioration of the immune response and the pro-inflammatory milieu related with components of the MetDiet may help to prevent or reduce the severity of COVID-19 disease, its role has still not been clarified. We think that a healthy dietary pattern, such as the MetDiet, may be a valuable supporting therapeutic strategy to improve the prognosis of individuals affected by infection of SARS-CoV-2, reducing the need to be treated in intensive care units.

Another aspect that needs to be taken into account is the obesity-dependent inflammation state [66]. In COVID-19 infectious disease, host factors determine disease severity and progression [100]. The major risk factors include male sex, age, smoking, obesity, and comorbid chronic diseases [101,103]. A very large amount of evidence suggests that age itself is the most significant risk factor for severe COVID-19 disease and its poor outcomes [104,105,106,107].

COVID-19, in its most severe form, causes a bilateral interstitial pneumonia that needs intensive care unit (ICU) ventilation support, and it is associated with a high mortality rate due to multi-organ failure. A smoking habit (e-cigarette, cigarette, or waterpipe) is a possible mode of transmission for SARS-CoV-2 for both active and passive smokers because it transmits salivary droplets into the surrounding environment and contaminates surfaces [108]. Smoking has been associated with rapid disease advancement [109,110]. In fact, smokers have a higher risk of contracting the SARS-CoV-2 due to impaired immune function, reduced lung function, and increased mucosal permeability [111]. Some studies have suggested that active smokers have a greater risk of severe COVID-19 symptoms, to be admitted to the intensive care unit, and of mortality than non-smokers do [111,112,113]. This is because nicotine can affect the renin–angiotensin system through upregulation of the ACE2 receptor in pulmonary epithelial cells, resulting in increased susceptibility and progression of COVID-19 disease [111].

In addition to proper nutrition and smoking cessation, strong evidence supports that regular physical activity (PA) results in a wide range of beneficial effects on health. In particular, regular PA improves the immune system, and determines a lower incidence and mortality from diverse viral infections [114,115,116]. PA also reduces systemic inflammation [116], and increases lung capacity and muscle function [117]. Thus, regular PA could play an important role in mitigating the severity of the COVID-19 outcomes. It has been demonstrated that patients with COVID-19 who were doing regular physical activity had a lower risk of hospitalization (OR: 1.20; 95% CI: 1.10 to 1.32), admission to the intensive care unit (OR: 1.10; 95% CI: 0.93 to 1.29), and mortality (OR: 1.32; 95% CI: 1.09 to 1.60) due to COVID-19 compared with patients who were consistently inactive [118]. Thus, regular PA could be an auxiliary tool against SARS-CoV-2 infection [115]. Furthermore, to contain the spread of COVID-19, Governments decided on a more stringent containment measure: Lockdown. The Lockdown has determined a radical change in eating habits and lifestyles of the population, with an increase of sedentary behavior [119] and smoking [120].

Numerous studies, in fact, reported during the Lockdown a reduction in the consumption of fresh food, accompanied by minerals and vitamins deficit, including beta-carotene, vitamin C, and vitamin E antioxidants molecules, as well as an increase of high-caloric foods: the ‘comfort foods’ with weight gain in all age groups [121,122,123,124,125]. It is well known that sedentary subjects are at higher risk of inadequate consumption of nutrients than are others. It has been deduced that the regulation of various cellular pathways can be affected by a person’s diet, as one ingests a myriad of different substances, which can cause long-term effects and influence the development of certain illnesses, such as infectious diseases [62,63]. Strong evidence indicates that a diet such as MetDiet, which contains a sufficient consumption of proteins, fibers (from whole grains), micronutrients (zinc, selenium, and vitamins A, C, D, and E), antioxidants and PUFA, has a positive effect to prevent the burden of major chronic disease conditions [23,24,25,26,27,28].

Some limitations need to be addressed. First, to date, there are no studies that have demonstrated the effectiveness of the MetDiet in preventing COVID-19 disease and reducing COVID-19-related clinical outcomes. Second, epidemiological analyses based on diagnostic cases are distorted by the diagnostic protocols and reports in each country, as well as by the pool of asymptomatic cases; any attempt to improve the diagnostic rate requires an economic, infrastructural, and logistic effort that is not always possible in the various European countries [126]. Third, MetDiet includes numerous nutrients that are also present in other eating patterns; however, a recent meta-analysis of randomized controlled trials that analyzed the effects of dietary patterns on biomarkers of immune responses and inflammation demonstrated that the MetDiet was the dietary pattern that showed the most prominent reductions of inflammatory biomarkers ([mean difference (MD): −1.07 pg/mL (95% CI: −1.94, −0.20); I2: 96%], IL-1β [MD: −0.46 pg/mL (95% CI: −0.66, −0.25); I2: 0%], and PCR [MD: −1.00 mg/L (95% CI: −2.02, 0.01); I2: 100%]) [127]. No solid effects were seen for the other dietary patterns included in the study, such as the Dietary Adherence to Stop Hypertension diet, and the vegetarian or vegan diets [127]. Fourth, it is important to note that adherence to the MetDiet has also decreased in recent years in the countries of the Mediterranean basin [35].

## 4. Conclusions

The MetDiet represents a precious dietary model for the prevention and treatment of chronic diseases such as obesity and metabolic syndrome; however, cultural and social changes in the world have caused a progressive abandonment of it and a simultaneous shift to the Western dietary pattern. This worrying phenomenon is particularly pronounced in the elderly population and in the countries of the Mediterranean basin. In summary, in a situation where reduction of susceptibility to SARS-CoV-2 virus in the general population is required, it may be paramount to follow the advice to adhere to the MetDiet, encouraging the consumption of food rich in nutrients with antioxidants and anti-inflammatory activities. The daily consumption of legumes, fruits, vegetables, and EVOO can be easily followed even in emergency conditions. We reviewed the most important literature focusing on the potential benefits of nutrients, vitamins, and components with anti-inflammatory and antioxidant activities, which may play a vital role in reducing susceptibility to developing viral infections during this time of global pandemic in all populations. MetDiet can be considered a dietary pattern that is naturally supplemented and that can reduce susceptibility to SARS-CoV-2, in association with nonsmoking and regular physical exercise. Therefore, the MetDiet could be considered a useful dietary option during the global pandemic of SARS-CoV-2 infection. However, we encourage future studies to corroborate the benefits of the MetDiet with regard to infection with SARS-CoV-2 in subjects without severe disease.

## Figures and Tables

**Table 1:** Possible anti-SARS-CoV-2 effects attributed to MetDiet.

Effects	Components	Food Sources	References
Lower Inflammation (CRP, IL-6, TNF-alpha, ROS, RNS)	PUFA, MUFA, polyphenols, antioxidants, fibers, vitamins, minerals	Fish, EVOO, fruit, vegetables, legumes, wine, whole grains	[16,20,23,24,25,26,27,28,29,30,31,32,33,34,35,36,37,38,39,40,41,42,43,44,45,46,47,48,49,50,51,52,53,54,55,56,57,58,59,60,61,62,63,64,65,66,67,68,69,70]
Boost Immune system (anti-thrombotic, anti-PAF effect) and antiviral effects (NF-κB, AP-1)	Vitamin A, C, E, D, selenium, zinc, phytochemicals, and omega-3 PUFA, polifenols, antioxidants, resveratrol,	Legumes, vegetables, fruit, EVOO, seeds, bran, nuts and dried fruit, shellfish, beef, tea, red wine	[14,42,48,51,52,53,57,58,59,70,71,72,73,74,75,76,77,78,79,80,81,82,83,84,85,86,87,88]
Boost Intestinal Barrier Function (gut microbiota)	Prebiotic substances, galactans, fructans, fibers, and inulins	Legumes, vegetables, fruit, nuts, seeds, bran, milk and yogurt	[46,47,48,64,65,72,89,90,91,92,93,94,95]
Improvement of the metabolic setting (ACE2, Leptin)	PUFA, MUFA, polyphenols, antioxidants, fibers, vitamins, minerals, prebiotic substances, polifenols, antioxidants, resveratrol	Legumes, vegetables, fruit, EVOO, seeds, bran, nuts and dried fruit, shellfish, beef, tea, red wine	[13,14,15,16,17,18,19,20,21,22,96,97,98,99,100,101]

Abbreviations: CRP, C-reactive protein; IL-6, interleukin-6; TNF-alpha, tumor necrosis factor-alpha; ROS, reactive oxygen species; RNS, reactive nitrogen species; PUFA, polyunsaturated fatty acids; EVOO, extra virgin olive oil.

## Data Availability

The data presented in this study are available on request from the corresponding author.
